# Relationship Between Diabetes, Stress, and Self-Management to Inform Chronic Disease Product Development: Retrospective Cross-Sectional Study

**DOI:** 10.2196/20888

**Published:** 2020-12-23

**Authors:** Jessica S Yu, Tong Xu, Roberta A James, Wei Lu, Julia E Hoffman

**Affiliations:** 1 Livongo Health Mountain View, CA United States

**Keywords:** diabetes mellitus, behavioral health, mental health, stress, technology

## Abstract

**Background:**

Technology is rapidly advancing our understanding of how people with diabetes mellitus experience stress.

**Objective:**

The aim of this study was to explore the relationship between stress and sequelae of diabetes mellitus within a unique data set composed of adults enrolled in a digital diabetes management program, Livongo, in order to inform intervention and product development.

**Methods:**

Participants included 3263 adults under age 65 who were diagnosed with diabetes mellitus and had access to Livongo through their employer between June 2015 and August 2018. Data were collected at time of enrollment and 12 months thereafter, which included demographic information, glycemic control, presence of stress, diabetes distress, diabetes empowerment, behavioral health diagnosis, and utilization of behavioral health-related medication and services. Analysis of variance and chi-square tests compared variables across groups that were based on presence of stress and behavioral health diagnosis or utilization.

**Results:**

Fifty-five percent of participants (1808/3263) reported stress at the time of at least 1 blood glucose reading. Fifty-two percent of participants (940/1808) also received at least 1 behavioral health diagnosis or intervention. Compared to their peers, participants with stress reported greater diabetes distress, lower diabetes empowerment, greater insulin use, and poorer glycemic control. Participants with stress and a behavioral health diagnosis/utilization additionally had higher body mass index and duration of illness.

**Conclusions:**

Stress among people with diabetes mellitus is associated with reduced emotional and physical health. Digital products that focus on the whole person by offering both diabetes mellitus self-management tools and behavioral health skills and support can help improve disease-specific and psychosocial outcomes.

## Introduction

Diabetes mellitus currently affects over 10.5% of the United States population, or over 34 million Americans, and comes with significant medical, psychological, and economic burdens [1]. It is the leading cause of kidney failure, nontraumatic lower limb amputations, and blindness; a major cause of cardiovascular disease and stroke; and the seventh leading cause of death among adults in the United States [2]. It is also related to behavioral health concerns. People with diabetes mellitus are two to three times more likely to be diagnosed with major depressive disorder than people without diabetes mellitus and have a 20% greater prevalence of anxiety disorders than those without [3,4]. The economic burden of diabetes mellitus includes cost to the individual as well as that to their employers and the healthcare system. People with diabetes mellitus spend an average of US $13,700 annually on medical expenses, which is more than double that of people without [5]. The US healthcare system spends an average of US $245 billion annually on diabetes, including expenses related to disability, work loss, and premature mortality [5].

Diabetes mellitus is a chronic and challenging illness, which requires patients to engage in complex, lifelong self-management of their condition. For an individual with diabetes mellitus, effective self-management is a daily routine of healthy eating, exercising, self-monitoring of blood glucose, adhering to medications, problem solving, reducing the risk of diabetes mellitus-related complications, and practicing healthy coping skills [6]. Managing these varied tasks can be a stressful experience, such that 18%-35% of people with diabetes mellitus experience high levels of “diabetes distress” or “significant negative emotional reactions to the diagnosis of diabetes, threat of complications, self-management demands, unresponsive providers, and/or unsupportive interpersonal relationships” [7]. Additionally, people with diabetes mellitus experience stress that is not condition related, just as people without diabetes mellitus do. This short- or long-term stress can come in many different forms such as, but not limited to, daily hassles, chronic stress, interpersonal stress, or work stress. Several studies have found associations between stress, self-management, and blood glucose control in people with diabetes mellitus. People who have coping deficits or who have high disease-related distress are less likely to engage in self-management behaviors, more likely to exhibit poor blood glucose control, and more likely to be at greater risk for diabetes complications [7,8].

Given the relationships between stress, self-management, and blood glucose control in people with diabetes mellitus, clinicians and researchers have long been interested in developing and refining methods to measure and treat stress. With regards to measuring stress, ecological momentary assessment is one now well-established method. Ecological momentary assessment involves the real-time collection of an individual’s in-the-moment thoughts, feelings, and behaviors, typically in the convenience of their natural environment [9]. This is in contrast to older, more traditional methods of measuring behavioral constructs that require individuals to recall past activity or mood. The advantages of ecological momentary assessment in the measurement of stress include more accurate assessment and a finer understanding of the way in which stress unfolds [9]. With regards to treating stress, several interventions exist that empower patients with the skills and tools to manage their emotional, mental, and physical health. Traditionally, these interventions have taken the form of in-person diabetes mellitus self-management education and support programs or in-person stress management training. Examples include Funnell et al’s [10] empowerment-based program, Hill-Briggs et al’s [11] problem-solving approach, Lorig’s [12] Chronic Disease Self-Management Program, and Surwit’s [13] stress management program. As technology has become increasingly ubiquitous, internet and mobile phone–based interventions have also been developed to deliver diabetes mellitus self-management education and support, of which many include stress management training [14].

However, both ecological momentary assessment and diabetes mellitus stress management interventions have largely been confined to research environments. Although numerous studies have shown them to be effective in measuring psychological constructs, increasing participants’ diabetes mellitus knowledge and self-care, improving blood glucose control and other physical health measures, and increasing quality of life, there is still a need to understand the real-world needs of people who are using technology to improve their health [12,15-18]. Currently, a search for the keyword “diabetes” in the Apple App Store alone produces hundreds of potential apps, yet it is unclear how many of these apps have been developed with a nuanced understanding of the day-to-day needs of the people with diabetes mellitus. Thus, the aim of the current study was to explore the relationship between stress and sequelae of diabetes mellitus using a unique and rich data set comprised of adults enrolled in a digital diabetes mellitus management program, Livongo, in order to inform intervention and product development.

## Methods

### Product and Participants

Livongo, a digital health company based in Mountain View, California, offers the Livongo for Diabetes Program as a benefit to employees at select US-based companies. The program provides members with (1) a cellular technology-enabled, two-way messaging device that measures blood glucose, stores blood glucose and contextual data, and feeds relevant algorithmic messages back to the individual; (2) unlimited blood glucose test strips; and (3) access to a team of certified diabetes educators for text or phone-based coaching (see Multimedia Appendices 1-3). In-depth overviews of the Livongo for Diabetes program and its efficacy on improving diabetes-related outcomes are available elsewhere [19-21]. Consistent with ecological momentary assessment methodology, each time participants completed a blood glucose check, participants were prompted to report their emotional or mental state by choosing one of the following feelings tags: “I feel fine,” “I don’t feel well,” “Light-headed,” “Stressed out,” “After exercise,” “Ate more,” “Increased meds,” “Missed meds,” or “Other.” Participants were not prompted to report their emotional or mental state in the absence of a blood glucose check.

Participants were adults under age 65 with diabetes mellitus who had access to Livongo through their employers between June 2015 and August 2018. A retrospective cross-sectional study was conducted with data collected while participants engaged in the Livongo for Diabetes Program. Medical and pharmacy claims data were obtained for participants for 12-month prestudy and poststudy index date to determine presence of behavioral health conditions. A participant’s study index date was determined as the first date with “Stressed out” reported during a blood glucose check. For participants without selection of stress during a blood glucose check, the study index date was set as the first blood glucose check. The data were used to ascertain whether participants were diagnosed with a behavioral health condition or received behavioral health treatment during the 24-month study period. Diagnoses were included based on ICD-10 (International Classification of Diseases, 10th Revision) codes corresponding to indexes F00-F99, “Mental, Behavioral, and Neurodevelopmental Disorders,” as well as procedures with current procedural terminology codes corresponding to therapy, assessment, and other related psychiatric procedures. Pharmacy claims used National Drug Code codes to identify drugs used to treat behavioral health conditions. Participants were grouped into 4 categories identifying the presence and absence of ecological momentary assessment of stress and behavioral health diagnosis/treatment.

The study received institutional review board approval from Aspire IRB for waiver of informed consent and full waiver of Health Insurance Portability and Accountability Act authorization (Protocol-LDR-2016). All procedures performed in studies involving human participants were in accordance with the ethical standards of the institutional research committee and with the 1964 Helsinki declaration and its later amendments.

### Measures

#### Demographic Information

Demographic data collected at the time of enrollment in Livongo included age, gender, race, ethnicity, body mass index, age first diagnosed with diabetes mellitus, number of years living with diabetes mellitus, and insulin use. Income and education level were estimated based on the American Census Survey, specifically by gathering the mean/median values within a participant’s zip code. This method has previously been used in public health research when self-report data on socioeconomic status are unavailable [22].

#### Glycemic Control

Participants self-reported hemoglobin A_1c_ (HbA_1c_) at time of enrollment. They then used their FDA-cleared, cellular-enabled glucometer to conduct blood glucose readings as desired or needed. For the purposes of this study, blood glucose data were collected for 12 months after the study index date.

#### Diabetes Distress

Participants completed the 2-item Diabetes Distress Scale at time of enrollment [23]. This scale screens for the presence of diabetes distress by asking respondents to rate on a scale of 1 to 6 (1=not a problem; 6=a very serious problem) the degree to which they are bothered by 1) “feeling overwhelmed with the demands of living with diabetes” and 2) “feeling that [they] are often failing with their diabetes routines.” The total score is calculated by adding the 2 responses. The 2-item Diabetes Distress Scale has been shown to have high sensitivity and specificity [23].

#### Diabetes Empowerment

Participants completed the 8-item Diabetes Empowerment Scale at time of enrollment [24]. The Diabetes Empowerment Scale assesses self-efficacy in people with diabetes mellitus. Respondents are asked to rate on a scale of 1 to 5 (1=strongly disagree; 5=strongly agree) the degree to which they agree with statements such as “In general, I believe I am able to turn my diabetes goals into a workable plan,” “I can ask for support for having and caring for my diabetes when I need it,” and “I know enough about myself as a person to make diabetes care choices that are right for me.” The total score is calculated by summing all responses and dividing by 8. The Diabetes Empowerment Scale is shown to have high reliability and content validity [24].

### Statistical Analyses

To explore the relationship between stress and sequelae of diabetes mellitus among participants using a real-world digital health product, statistical analyses were conducted for primarily descriptive purposes. Demographics, participant characteristics, Diabetes Empowerment Scale scores, and Diabetes Distress Scale scores at enrollment and blood glucose control were compared by reported stress and behavioral health diagnosis/utilization categories. Analyses of variance and 2-tailed *t* tests were used for between group comparisons of continuous variables, and Chi-square test was used for categorical variables group comparisons. The proportion of members with behavioral health diagnosis, procedure, and pharmacy in the pre- and post-study periods was compared for participants with and without stress with McNemar’s test. *P* values less than .05 were considered statistically significant. No adjustments for multiple comparisons were made.

## Results

Of the 29,270 individuals with diabetes mellitus that enrolled in the Livongo for Diabetes program and received devices, 25,286 (86.3%) engaged with the program by taking at least one blood glucose reading. Of those, 18,398 (72.8%) remained active for 12 months. Approximately 24% of 12-month active individuals (4473/18,398) were linked to available claims data. There were 3263 participants with 24 months of continuous medical and pharmacy claims in the Livongo Diabetes Program during the study period. Their mean age was 51.1 (SD 10.0) years; 1602 (49.1%) were male; 1950 (59.8%) were non-Hispanic; and 1683 (51.6%) were Caucasian. Table 1 contains additional overall population demographics and characteristics arranged by combinations of the presence of stress and behavioral health diagnosis or treatment.

**Table 1:** Participant demographics and characteristics.

Characteristic		Total sample (N=3263)		Stress, no behavioral health diagnosis/treatment (n=868)		Stress, with behavioral health diagnosis/treatment (n=940)		No stress, with behavioral health diagnosis/treatment (n=538)		No stress, no behavioral health diagnosis/treatment (n=917)			*P* value^a^	
Age, years (SD)		51.1 (10.0)		50.6 (10.3)		51.2 (9.8)		51.6 (9.5)		51.0 (10.3)			.28	
**Gender, n (%)**												**.35**		
	Male		1,602 (49.1)		393 (45.3)		376 (40.0)		264 (49.1)		569 (62.1)			
	Female		1,661 (50.9)		475 (54.7)		564 (60.0)		274 (50.9)		348 (37.9)			
**Ethnicity, n (%)**												**.36**		
	Hispanic		519 (15.9)		156 (18.0)		138 (14.7)		80 (14.9)		145 (15.8)			
	Non-Hispanic		1,950 (59.8)		492 (56.7)		573 (61.0)		335 (62.3)		550 (60.0)			
	Unknown		794 (24.3)		220 (25.3)		229 (24.4)		123 (22.9)		222 (24.2)			
**Race, n (%)**												**<.001**		
	Caucasian		1,683 (51.6)		418 (48.2)		556 (59.1)		308 (57.2)		401 (43.7)			
	Black/ African American		385 (11.8)		119 (13.7)		87 (9.3)		57 (10.6)		122 (13.3)			
	Asian/ Chinese/ Japanese/ Korean		208 (6.4)		51 (5.9)		20 (2.1)		28 (5.2)		109 (11.9)			
	Native Hawaiian/ Pacific Islander		8 (0.2)		2 (0.2)		3 (0.3)		3 (0.6)		0 (0)			
	American Indian/ Alaskan/ Native		19 (0.6)		2 (0.2)		10 (1.1)		2 (0.4)		5 (0.5)			
	Latino/ Mexican		1 (0.0)		0 (0)		0 (0)		0 (0)		1 (0.1)			
	Other		327 (10.0)		115 (13.2)		75 (8.0)		38 (7.1)		99 (10.8)			
	Unknown		632 (19.4)		161 (18.5)		189 (20.1)		102 (19.0)		180 (19.6)			
Annual income, US $, mean (SD)		72,946.87 (25,464.50)		72,164.70 (25,382.00)		71,598.80 (24,102.90)		72,332.60 (24,482.60)		75,429.50 (27,577.40)			.008	
**Education Level, n (%)**														
	High school		922 (28.3)		246 (28.3)		270 (28.7)		155 (28.9)		251 (27.4)			.008
	Bachelor's degree		584 (17.9)		153 (17.6)		166 (17.7)		95 (17.6)		170 (18.6)			.08
	Graduate degree		316 (9.7)		82 (9.4)		90 (9.6)		53 (9.8)		91 (9.9)			.505
**Diabetes mellitus type, n (%)**												**.10**		
	Type 1		421 (12.9)		126 (14.5)		108 (11.5)		53 (9.9)		134 (14.6)			
	Type 2		2,835 (86.9)		740 (85.3)		830 (88.3)		484 (90.0)		781 (85.2)			
	Unknown		7 (0.2)		2 (0.2)		2 (0.2)		1 (0.2)		2 (0.2)			
Duration of illness, years, mean (SD)		8.4 (8.1)		8.6 (7.8)		9.0 (8.6)		8.3 (7.8)		7.8 (7.9)			.009	
**Insulin use, n (%)**												**<.001**		
	No		2,208 (67.7)		552 (63.6)		622 (66.2)		363 (67.5)		671 (73.2)			
	Once per day		428 (13.1)		131 (15.1)		113 (12.0)		77 (14.3)		107 (11.7)			
	Less than once per day		627 (19.2)		185 (21.3)		205 (21.8)		98 (18.2)		139 (15.2)			
Blood glucose value over 12 months, mg/dL, mean (SD)		150.1 (44.1)		154.5 (43.9)		152.8 (42.9)		145.7 (43.4)		145.9 (46.1)			<.001	
**Percent time in specified range over 12 months, mean (SD)**														
	Less than 54 mg/dL		1.08 (4.00)		1.06 (3.23)		0.85 (2.89)		1.50 (6.24)		1.09 (4.47)			.04
	55-70 mg/dL		1.89 (3.86)		1.85 (3.36)		1.79 (3.36)		2.26 (5.47)		1.82 (3.89)			.13
	71-180 mg/dL		74.1 (25.6)		72.1 (25.9)		72.9 (25.2)		75.5 (25.6)		76.5 (27.0)			.001
	More than 180 mg/dL		22.9 (25.5)		25.0 (25.5)		24.5 (25.0)		20.7 (24.6)		20.6 (26.4)			<.001
DDS^b^ score, mean (SD)		2.38 (1.18)		2.47 (1.21)		2.77 (1.26)		2.18 (1.09)		2.03 (1.13)			<.001	
DES^c^ score, mean (SD)		3.84 (0.77)		3.84 (0.84)		3.67 (0.74)		3.94 (0.67)		3.94 (0.80)			.001	
Body mass index, mean (SD)		33.4 (7.4)		33.3 (7.4)		34.7 (7.7)		33.5 (7.7)		32.0 (7.1)			<.001	

^a^Continuous variable were compared using analysis of variance; categorical variables were compared using chi-square test.

^b^DDS: Diabetes Distress Scale.

^c^DES: Diabetes Empowerment Scale.

Of the 3263 participants with 24 months of continuous medical and pharmacy claims enrolled in the Livongo Diabetes Program during the study period, 1808 (55%) reported feeling “stressed out” at the time of at least 1 blood glucose reading. Participants who scored higher on the 2-item Diabetes Distress Scale, lower on the Diabetes Empowerment Scale, and had greater insulin use at enrollment were more likely to report feeling stressed on at least 1 blood glucose reading (2-item Diabetes Distress Scale score: 2.61.25 vs 2.091.11, respectively, *P*<.001; Diabetes Empowerment Scale score: 3.80.79 vs 3.90.79, respectively, *P*<.001; insulin: 35.1% vs 28.9%, respectively, *P*<.001). Participants who reported feeling stressed were also more likely to exhibit poorer blood glucose control throughout the study (proportion of blood glucose readings >180 mg/dL=0.250.25 vs 0.210.26, respectively, *P*<.001).

Of the 1808 participants who reported feeling stressed at the time of at least 1 blood glucose reading, 940 (52%) also received at least one behavioral health diagnosis or intervention during the study period. Compared to participants who neither reported stress nor received a behavioral health diagnosis/intervention, these participants had higher body mass index (34.77.67 vs 32.87.36, respectively, *P*<.001) and duration of illness (9.08.60 years vs 8.27.86 years, respectively, *P*=.008) at enrollment.

**Figure 1:**
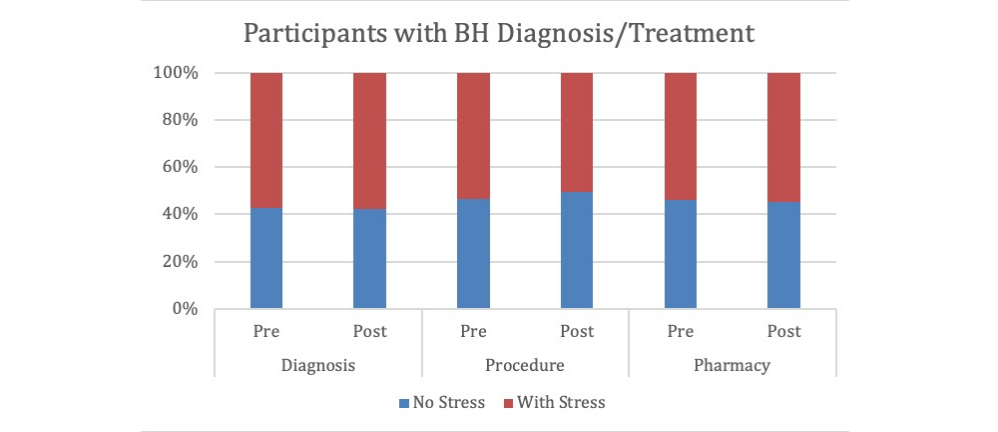
Participants with behavioral health diagnosis/treatment. BH: Behavioral health; Pre: 12 months prior to the study index date; Post: 12 months following the study index date.

Table 2 contains the blood glucose checking frequency and values during the 12-month period following the study index date. Participants who reported stress started with higher A_1c_ at enrollment and continued to exhibit poorer glycemic control throughout the study (proportion of blood glucose readings >180 mg/dL=0.250.25 vs 0.210.26, respectively, *P*<.001). The presence or absence of behavioral health diagnosis/treatment did not appear to impact blood glucose checking or glucose management. Participants had similar behavioral health diagnoses, procedures, and treatments across the 4 groups. No significant differences were observed. However, participants with stress had more diagnoses and treatments than participants without stress (see Figure 1).

**Table 2:** Blood glucose checking frequency and values during the 12-month period following study index date.

Characteristic		Stress, no behavioral health diagnosis/treatment (n=868)	Stress, with behavioral health diagnosis/treatment (n=940)	No stress, with behavioral health diagnosis/treatment (n=538)	No stress, no behavioral health diagnosis/treatment (n=917)	*P* value^a^
A_1C_ at enrollment, mean (SD)		7.8 (1.8)	7.6 (1.7)	7.6 (1.7)	7.5 (1.7)	.02
Number of stressed blood glucose checks, value (SD)		9.52 (29)	12.7 (39.1)	N/A^b^	N/A	<.001
Total number of blood glucose checks, value (SD)		329.2 (399.8)	314.6 (406.4)	193.5 (273.6)	203.4 (275.9)	<.001
Blood glucose, values (SD)		154.5 (43.9)	152.8 (42.9)	145.7 (43.4)	145.9 (46.1)	<.001
Maximum blood glucose, value (SD)		322.8 (121.5)	317.9 (126.8)	279.2 (116.6)	274.2 (114.5)	<.001
Glycemic variability, value (SD)		28.8 (11.7)	28.3 (11)	27.7 (12.6)	26 (12.5)	<.001
**Percent time in specified range over, value (SD)**						
	Less than 54 mg/dL	1.1 (3.2)	0.9 (2.9)	1.5 (6.2)	1.1 (4.5)	.04
	55-70 mg/dL	1.9 (3.4)	1.8 (3.4)	2.3 (5.5)	1.8 (3. 9)	.13
	71-180 mg/dL	72.1 (25.9)	72.9 (25.2)	75.5 (25.6)	76.5 (27.0)	.001
	More than 180 mg/dL	25.0 (25.5)	24.5 (25.0)	20.7 (24.6)	20.6 (26.4)	<.001

^a^Analysis of variance was used for between group comparisons.

^b^N/A: not applicable.

## Discussion

### Overview

The purpose of this descriptive study was to explore the relationships between stress, diabetes mellitus–related symptoms, and blood glucose control among people using Livongo, a digital diabetes mellitus management program for people with diabetes mellitus and other chronic conditions. The study was unique in that participants were people with diabetes mellitus using technology in their everyday lives, ecological momentary assessment of stress at the time participants checking their blood glucose was included, and healthcare utilization data to track behavioral health diagnoses and treatment were reviewed.

### Principal Results

Approximately half of participants reported experiencing stress. Additionally, stress was related to greater diabetes mellitus distress, lower diabetes mellitus empowerment, greater insulin use, and poorer glycemic control. Over half of participants who experienced stress also had at least one behavioral health diagnosis or received some kind of behavioral health intervention, which was related to greater body mass index and longer duration of illness. In other words, stress among people with diabetes mellitus is associated with reduced emotional and physical health.

### Limitations

This study has several strengths and weaknesses. Although participants self-reported clinical data such as A_1c_ at time of enrollment, stress data were collected noninvasively and in real-time. This enabled more accurate assessment of stress, although more analysis is required to evaluate stress intensity and duration on clinical outcomes and association with behavioral health. Further, a large sample size of people with both type 1 and type 2 diabetes mellitus, some of whom had been recently diagnosed and others who had lived with diabetes mellitus for several years, and some of whom reported varying degrees of control, provided an increased generalizability of results.

The study was limited by the single-item ecological momentary assessment prompt following blood glucose measurements, which only allowed one response for a variety of correlated feeling and nonfeeling state constructs (eg, stress and missing medications; exercise and feeling fine) [25]. Although individuals were instructed to select the item they believed best described how they felt at the time, the number of reports of stress were likely undercounted if individuals believed some other item applied to their current situation. Future work could use previous literature to inform separate ecological momentary assessment items, such as using single-item stress measures derived from the Perceived Stress Scale [26]. The study may also have been limited by the use of zip code–based socioeconomic status estimation. Although this method is common in public health research [22], it is important to acknowledge that socioeconomic status itself is known to be related to behavioral and physical health [27]. Therefore, the absence of self-report socioeconomic status data may limit our ability to interpret results.

### Comparison With Prior Work

Regardless of the study’s limitations, our findings are important in the current context of diabetes mellitus. Despite our nation’s tremendous efforts to prevent the disease, its prevalence continues to rise. Experts project that by 2030, diabetes mellitus will affect nearly 55 million Americans, be attributable to 385,800 deaths per year, and cost the US healthcare system US $622 billion [28]. Therefore, it is ever more imperative to find ways to help people with diabetes mellitus manage the emotional, mental, and physical toll of the disease.

Technology, particularly smartphone technology, can be incredibly useful in diabetes mellitus management. Mobile apps can enable people with diabetes mellitus to track their eating, physical activity, and medication use. They can also deliver diabetes mellitus education and support via written content, online chat groups, and health coaching. Cellularly-enabled glucose meters can provide people with real-time feedback on their glycemic control that they can share with their providers.

Digital products offering a flexible, person-centered approach including diabetes mellitus self-management tools and behavioral health skills and support may exhibit the most promise in improving disease-specific and psychosocial outcomes. Ideally, such products would offer a full spectrum of services to enhance a person’s emotional, mental, and physical health. The ideal product would include elements of both collaborative and stepped care, such as screening and assessment, self-help content, guided self-help content with access to coaches to pace the individual or respond to the individual’s questions, the opportunity to receive individual treatment from a provider, the opportunity for providers to work with one another in service of the individual’s care, and access to a peer community. Research has shown that collaborative and stepped care models offer high levels of patient satisfaction, can help reduce drop out from treatment, are as clinically effective as usual treatment, and are cost effective [29-33]. The ideal product would also be multimodal and highly personalized, offering access via computer, phone, and video in an integrated approach to care when a person is most desirous or in need of care, for instance, when a person has endorsed high stress at the time of a blood glucose check.

### Conclusions

In conclusion, this study highlights the relationship between diabetes mellitus and stress in a real-world context. While this finding is not novel, rapid advancements in technology are advancing our ability to assess and treat a myriad of health concerns in real-time in the context of an individual’s life. This increased accuracy and timeliness represents an important step forward in the depth of understanding of this connection. Clinicians, researchers, product developers, software engineers, and other technology experts must come together to create clinically effective, cost effective, exciting, and engaging products to help people optimize all aspects of their health.

## Appendix

Multimedia Appendix 1 Real-Time Tags to Capture Context.

Multimedia Appendix 2 Real-Time Analytics and Feedback for Blood Glucose Checks.

Multimedia Appendix 3 Health Nudges, Engagement Powered by Machine Learning.
