# Supplementary figures and images for: Relationship Between Diabetes, Stress, and Self-Management to Inform Chronic Disease Product Development: Retrospective Cross-Sectional Study
Source: JMIR Diabetes. 2020 Dec 23;5(4):e20888. doi: 10.2196/20888 (PMC7787890; doi:10.2196/20888)

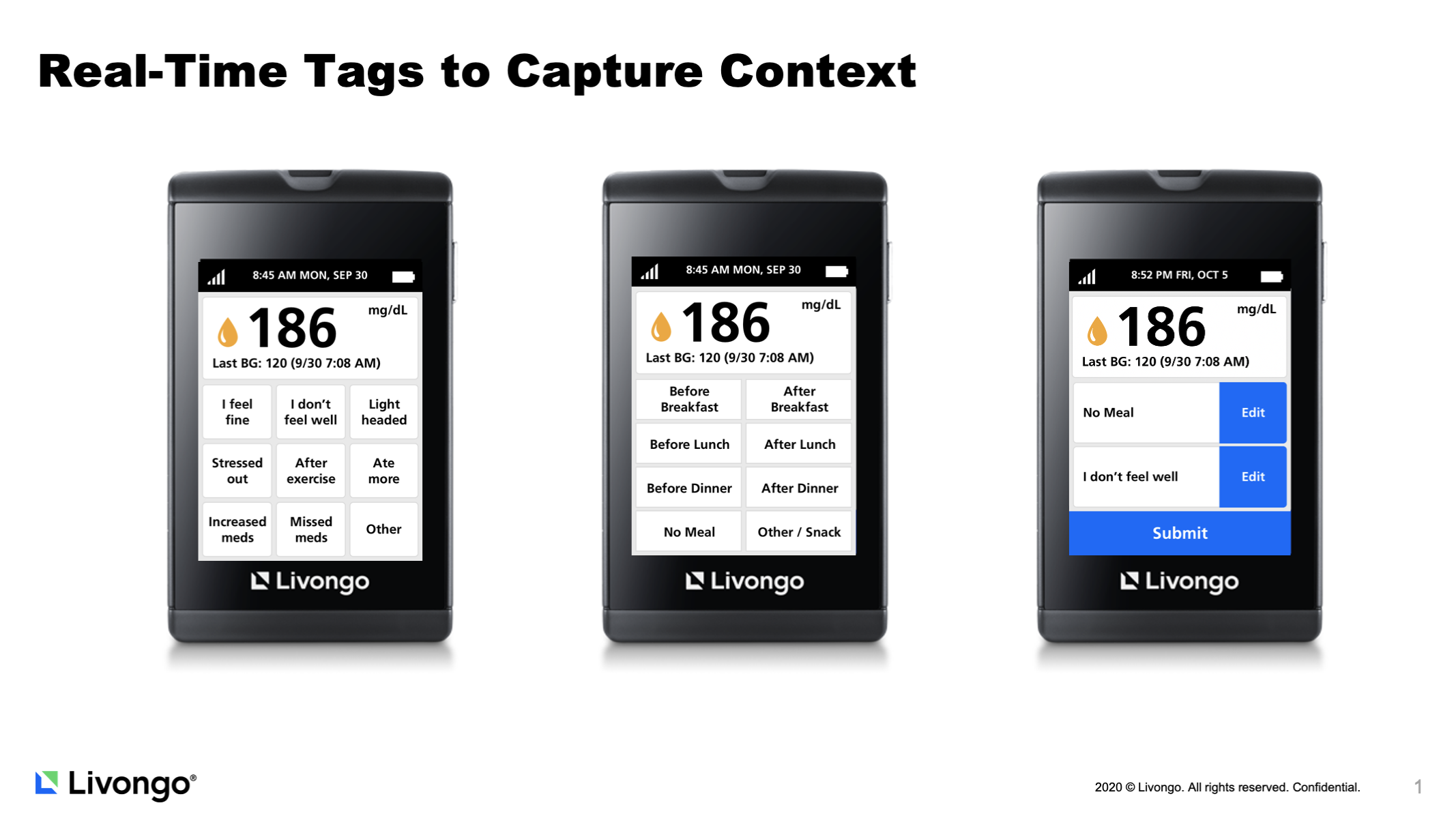

Supplement: Multimedia Appendix 1 [file diabetes_v5i4e20888_app1.png]

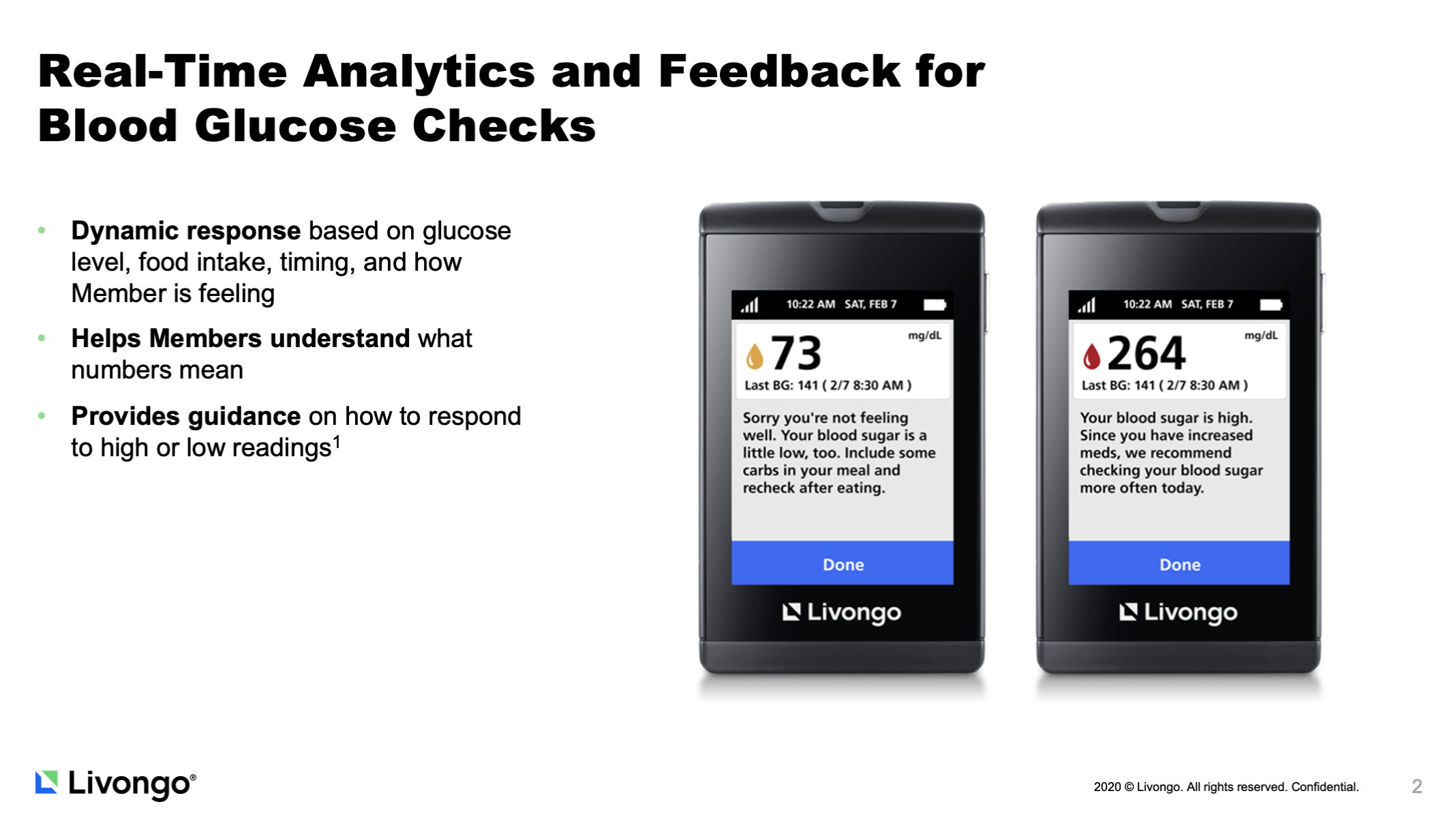

Supplement: Multimedia Appendix 2 [file diabetes_v5i4e20888_app2.png]

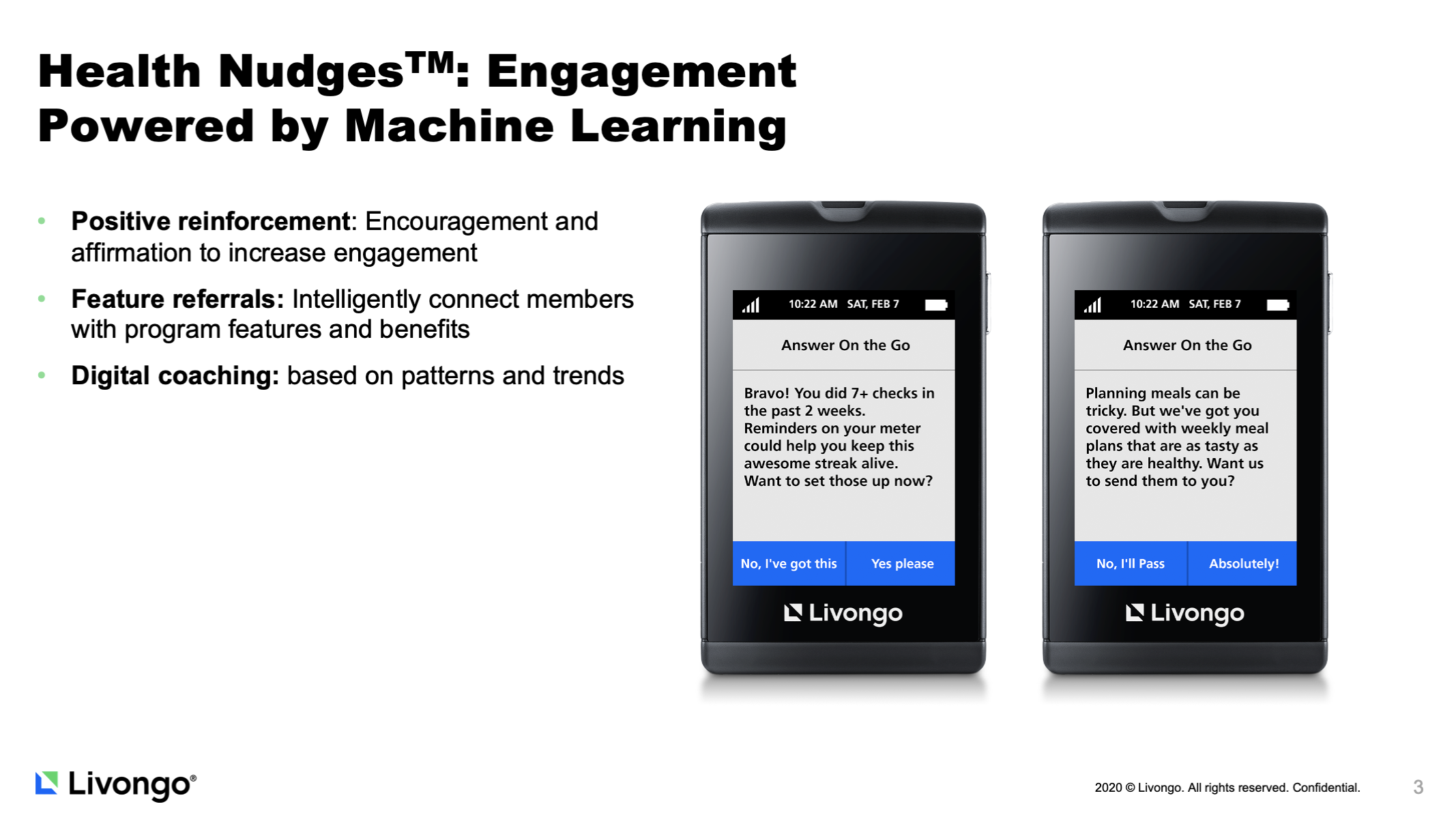

Supplement: Multimedia Appendix 3 [file diabetes_v5i4e20888_app3.png]
